# Influence of Chirality of Crizotinib on Its MTH1 Protein Inhibitory Activity: Insight from Molecular Dynamics Simulations and Binding Free Energy Calculations

**DOI:** 10.1371/journal.pone.0145219

**Published:** 2015-12-17

**Authors:** Yuzhen Niu, Dabo Pan, Danfeng Shi, Qifeng Bai, Huanxiang Liu, Xiaojun Yao

**Affiliations:** 1 State Key Laboratory of Applied Organic Chemistry and Department of Chemistry, Lanzhou University, Lanzhou, 730000, China; 2 School of Pharmacy, Lanzhou University, Lanzhou, 730000, China; 3 Key Lab of Preclinical Study for New Drugs of Gansu Province, Lanzhou University, Lanzhou, 730000, China; 4 The Separating Scientific Institute of Lanzhou, Lanzhou, 730000, China; University of Calgary, CANADA

## Abstract

As a promising target for the treatment of lung cancer, the MutT Homolog 1 (MTH1) protein can be inhibited by crizotinib. A recent work shows that the inhibitory potency of (S)-crizotinib against MTH1 is about 20 times over that of (R)-crizotinib. But the detailed molecular mechanism remains unclear. In this study, molecular dynamics (MD) simulations and free energy calculations were used to elucidate the mechanism about the effect of chirality of crizotinib on the inhibitory activity against MTH1. The binding free energy of (S)-crizotinib predicted by the Molecular Mechanics/Generalized Born Surface Area (MM/GBSA) and Adaptive biasing force (ABF) methodologies is much lower than that of (R)-crizotinib, which is consistent with the experimental data. The analysis of the individual energy terms suggests that the van der Waals interactions are important for distinguishing the binding of (S)-crizotinib and (R)-crizotinib. The binding free energy decomposition analysis illustrated that residues Tyr7, Phe27, Phe72 and Trp117 were important for the selective binding of (S)-crizotinib to MTH1. The adaptive biasing force (ABF) method was further employed to elucidate the unbinding process of (S)-crizotinib and (R)-crizotinib from the binding pocket of MTH1. ABF simulation results suggest that the reaction coordinates of the (S)-crizotinib from the binding pocket is different from (R)-crizotinib. The results from our study can reveal the details about the effect of chirality on the inhibition activity of crizotinib to MTH1 and provide valuable information for the design of more potent inhibitors.

## Introduction

MutT Homolog 1(MTH1), a nucleotide pool sanitizing enzyme, is a new therapeutic target in RAS-driven lung cancer reported recently [1]. MTH1 belongs to the Nudix hydrolase superfamily, characterized by a conserved 23-residue sequence segment (GX5EX7REUXEEXGU, U = I, L or V) [2]. MTH1 can implicate oncogenic KRAS-driven transformation of lung epithelial cells, evade oxidative DNA damage-mediated induction of cellular senescence, and maintain optimal oncogene levels in KRAS-mutant NSCLC cells that are refractory to senescence induction [3, 4]. Oncogenic KRAS can promote production of reactive oxygen (ROS) [5–7], which can attack almost all biological molecules, such as DNA and protein, and produce a variety of negative effects. Previous study has demonstrated that normal cells do not need MTH1, but cancer cells, due to high level of ROS, need MTH1 to survive [8]. Selective inhibition of MTH1 by small molecules leads to DNA damage and suppresses cancer growth effectively, thus revealing MTH1 as a promising target for anticancer therapies [1, 9].

By using a chemical proteomics strategy, Kilian and colleagues confirmed that the kinase inhibitor crizotinib can inhibit MTH1 at nanomolar level [1]. Crizotinib is an oral small-molecule inhibitor of anaplastic lymphoma kinase (ALK) approved by US Food and Drug Administration (FDA) for the treatment of advanced non-small cell lung cancer (NSCLC) with ALK rearrangements [10]. The study reported by Kilian *et al*. shows that the clinically used (R)-enantiomer of crizotinib is almost inactive (IC_50_ = 1375nM) but (S)-crizotinib is a low nanomolar MTH1 inhibitor (IC_50_ = 72nM) [1]. Understanding about how the chirality influences the binding between crizotinib and MTH1 should be valuable to elucidate the inhibition and enantiomer-selectivity mechanism, and further provide some clues for the design of more potent inhibitors of MTH1.

The efficacy of a drug is not only associated with thermodynamics but also related to the binding kinetics between the drug and a defined target. The thermodynamics method such as Molecular Mechanics/Generalized Born Surface Area (MM/GBSA) [11–14] can provide the information about the drug binding affinity, while the free energies calculated by the adaptive biasing force (ABF) [15] can provide information about ligand–receptor binding kinetics. The combination use of binding free energy calculations by MM/GBSA and ABF should provide much useful information to understand the inhibition and enantiomer-selectivity mechanism of MTH1. In this study, the molecular mechanism of the binding processes of (S)-crizotinib and (R)-crizotinib to MTH1 were studied by molecular dynamics (MD) simulations, Molecular Mechanics/Generalized Born Surface Area (MM/GBSA) free energy calculations. The adaptive biasing force (ABF) technique was further employed to elucidate the difference of the unbinding pathways of the (R)- and (S)-crizotinib from the binding pocket of MTH1. We expect that this work would provide more details about the influence of chirality of crizotinib on the MTH1 inhibition activity and provide valuable information for the future design of more potent selective MTH1 inhibitors.

## Materials and Methods

### Simulation systems preparation

The initial 3-D coordinates of the (S)-crizotinib/MTH1 and (R)-crizotinib/MTH1 complexes were retrieved from the Protein Data Bank (PDB ID: 4C9X and 4C9W) [1]. The piperidine of the Crizotinib molecule in the (R)-crizotinib/MTH1 complex (PDB ID: 4C9W) was built by overlaying the piperidine group of (S)-crizotinib onto the (R)-crizotinib. It is important to note that in the structure of (S)-crizotinib/MTH1 (PDB ID: 4C9X), the residue Asn33 has only one conformer. Since usually Asn has two conformers, we used the one that has the same conformation to the crystal conformer. The Phe27 of MTH1 in (R)-crizotinib/MTH1 complex (PDB ID: 4C9W) was built by the *Protein Preparation Wizard* in Schrodinger 2009 [16]. We also used *Protein Preparation Wizard* to add side chain of residues, hydrogen atoms, assign protonation states, and relax the amino residue side chains of the proteins. The partial charges of the inhibitors were derived by using the restrained electrostatic potential (RESP) [17–19] fitting procedure based on the electrostatic potentials calculated by Hartree-Fock (HF) method with 6-31G (d) basis set in the Gaussian09 package [20]. The values of partial charges for (S)-crizotinib, and (R)-crizotinib were listed in S1 Table and S2 Table. The general AMBER force field (GAFF) [21] and AMBER03 force field (ff03) [22] were used for the inhibitors and proteins, respectively. Then, the two starting structures were placed in an orthorhombic periodic box of TIP3P water molecules [23], with a separation margin from the solute of 10 Å in each dimension.

### Conventional molecular dynamics simulations

**Fig 1:**
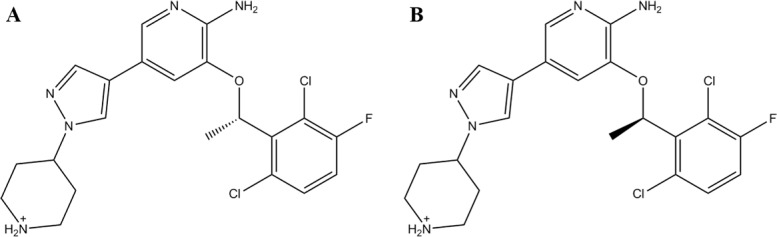
The structures of (S)-crizotinib (A) and (R)-crizotinib (B).

MD simulations of (S)-crizotinib (Fig 1A) and (R)-crizotinib (Fig 1B) in complex with MTH1 were performed by using NAMD 2.9 simulation package [24]. Long-range electrostatic interactions were handled by the Particle Mesh Ewald (PME) algorithm [25], while the short-range nonbonded interactions were calculated based on a cutoff of 10 Å. A steepest-descent minimization scheme was initially applied to the systems for 40000 steps, and then the systems were gradually heated in the NVT ensemble from 0 to 310 K in 100 ps by applying weak harmonic restraints with a constant force of 10 kcal/mol·Å^2^ on the C and N atoms of the protein backbone. Then, the restrain was gradually decreased within 0.9 ns from 10 to 0.01 kcal/mol·Å^2^. Finally, 20 ns MD simulations at a temperature of 310 K and a pressure of 1 atm. were carried out without any restrain. All bonds involving hydrogen atoms were restrained using the SHAKE [26] algorithm, and the time step was set to 2 fs.

### Binding free energy calculations

The binding free energies of (S)-crizotinib and (R)-crizotinib to the MTH1 protein were predicted by the MM/GBSA method [27] in AMBER10 [28, 29] since it gives better ranking capabilities for binding affinities than Molecular Mechanics/Poisson-Boltzmann Surface Area (MM/PBSA) for most cases [30–32]. The first step of MM/GBSA is to generate a number of snapshots extracted from the stable MD production trajectory of the complex. Here, 500 snapshots were extracted from the last 10ns MD trajectory (repeated sampling 10 times). For each snapshot, the ligand binding free energy is estimated by the following equation:

$$<\Delta G_{bind}>=<\Delta H_{MM}>+<\Delta G_{solvation}>-T<\Delta S_{MM}>$$ (1)

Where <Δ*G*_bind_ > is the calculated average free energy, and <Δ*E*_MM_ > is the average molecular mechanical energy.

$$<\Delta E_{MM}>=<\Delta E_{bond}>+<\Delta E_{angle}>+<\Delta E_{tors}>+<\Delta E_{vdW}>+<\Delta E_{elec}>$$ (2)

$$<\Delta G_{solvation}>=<\Delta G_{GB}>+<\Delta G_{SA}>$$ (3)

Where these correspond to the bond, angle, torsion, vander Waals, and electrostatic terms in the molecular mechanical force field evaluated with no nonbonded cutoff. <Δ*G*_solvation_ > is the desolvation free energy upon ligand binding, and it can be decomposed into polar (<Δ*G*_*GB*_>) and nonpolar contributions (<Δ*G*_*SA*_>); The polar contribution of desolvation (<Δ*G*_*GB*_>) was calculated based on Generalized Born (GB) model (igb = 2) [33]. The dielectric constants for solute and solvent were set to 1 and 80, respectively. The nonpolar contribution of desolvation (<Δ*G*_*SA*_>) was determined by solvent accessible surface area (SASA) using the LCPO method [34]: Δ*G*_SA_ = 0.0072 × ΔSASA. The conformational entropy contribution (−*T*<Δ*S*>) upon ligand binding was calculated using normal-mode analysis [35–38] in the AMBER program. The contributions of entropy to binding free energy arise from changes of the translational, rotational, and vibrational degrees of freedom, defined as follows

$$S=S_{translational}+S_{rotaional}+S_{vibrational}$$ (4)

In the normal-mode analysis, the frequencies of the normal modes are calculated from a molecular mechanics force field using the Hessian matrix of second energy derivatives in terms of the force constants for each type of interaction. All minimizations and normal-mode calculations were performed with a distance-dependent dielectric function 4Rij (the distance between two atoms) to mimic solvent screening. The structures were further minimized with no cutoff for nonbonded interactions by using conjugate gradient and then Newton–Raphson minimizations until the RMS of the elements in the gradient vector was less than 10^−4^ kcal/(mol·Å). Due to the high computational cost in the entropy calculation, only 50 snapshots were extracted from the last 5ns MD trajectory were used to calculate the entropic contribution.

### Per-residue free energy decomposition analysis

To identify the key residues responsible for the crizotinib binding process, the interaction between ligand and each residue was computed by using the MM/GBSA decomposition process in AMBER10 [28, 29]. All energy components were calculated using the 500 snapshots extracted from the last 5 ns MD trajectory. The binding interaction for each residue–inhibitor pair includes four terms: van der Waals contribution (<Δ*G*_vdW_>), electrostatic contribution (<Δ*G*_ele_>), polar solvation contribution (<Δ*G*_GB_>), and nonpolar solvation contribution (<Δ*G*_SA_>):

$$<\Delta G_{residue-inhibitor}>=<\Delta G_{vdW}>+<\Delta G_{ele}>+<\Delta G_{GB}>+<\Delta G_{SA}>$$ (5)

### Adaptive biasing force simulations

The adaptive biasing force(ABF) method [15, 39, 40] developed by Darve *et al*. has been widely used in identifying reaction path coordinates[41, 42] and calculating free energy[16, 42, 43]. To carry out ABF simulations, an external biasing force, estimated locally from the sampled conformations of the system, is applied at each step to facilitate the biased molecule in overcoming significant energy barriers along the reaction coordinate (RC). The advantage of ABF is that the sampling of an order parameter or a low-dimensional hyper surface becomes uniform rapidly, which in turn greatly improves the statistical precision of the calculated free energy. Therefore, in this work, we used the ABF method to explore the change of the potentials of mean force (PMF) of (S)-crizotinib and (R)-crizotinib escaped from the MTH1 active pocket along the RC (Z-axis). The PMF depth (ΔW_PMF_) [44–46], which can be obtained by ΔW_PMF-lowest_ -ΔW_PMF-highest_, was directly extracted from the ABF simulations based on 18-23Å of the reaction coordinates. The egress routes for (S)-crizotinib and (R)-crizotinib from the buried protein binding pocket of MTH1 were defined by the distance between N of Asp119 and C10 of (S)-crizotinib or (R)-crizotinib. In order to remove the effect of initial structures to the exit paths, five snapshot structures extracted from the last 5 ns equilibrium trajectories were used as the starting structures for the ABF simulations by using the same parameter set. The direction of the RC was rotated to the Z-axis. The initial structures were solvated again in a truncated octahedron box of TIP3P water molecules with a margin distance of 15Å. Prior to the ABF simulations, 1ns MD simulations were performed in the NPT ensemble (*P* = 1atm and *T* = 310*K*) to equilibrate the water molecules and ions with the receptor and ligand restrained with 1.0 kcal/mol.Å^2^. In the ABF simulations, the residues out of 10 Å of the ligand were restrained with 5 kcal/mol.Å^2^ to guarantee the direction of the RC. The length of the RC was separated into 23 windows with 1 Å/window and 0.1 Å/bin (10bin/window). Upper and lower wall constants were both set to 100 kcal/mol.A^2^ in high barrier regions and low barrier regions to ensure full sampling. The *fullsamples* parameter was set to 1000 at each window prior to biasing. 4 and 3 ns MD simulations were performed for each window, which guaranteed the reliability of predicting the unbinding pathway by using the ABF method and the convergence of the PMF as shown in S2 Fig. A total of 736 ns MD simulations were performed for the two systems (368 ns for each system).

## Results and Discussion

### Structural flexibility and stability of the simulation systems

**Fig 2:**
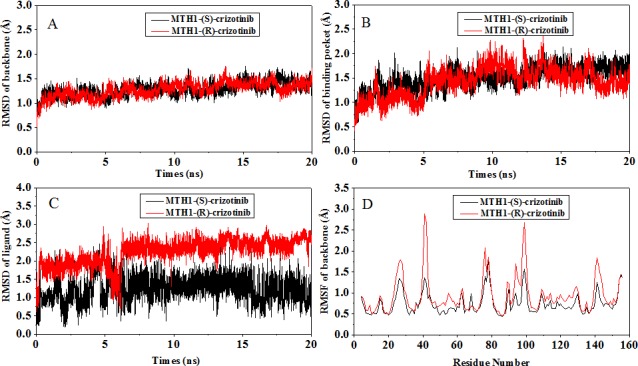
The monitoring of MD trajectories. (A) Time evolution of the RMSD of all protein backbone atoms; (B) Time evolution of the RMSD of Cα atoms for the residues around 5 Å of ligand; (C) Time evolution of the RMSD of heavy atoms for the ligand; (D) Time evolution of the RMSF of Cα atoms of the protein. The values reflect the equilibration of each of the systems relative to the initial structures.

The conformational stabilities of the (S)-crizotinib MTH1 and (R)-crizotinib MTH1 complexes were monitored by the root-mean-square deviation (RMSD) values of the C_α_ atoms of MTH1, the heavy atoms of the ligand and the root mean square fluctuations (RMSFs) relative to their initial minimized structures. As shown in Fig 2, after 7ns, the RMSDs of the both systems tend to converge [47], indicating that the systems are stable and equilibrated. The (R)-crizotinib/MTH1 complex shows higher fluctuations than the (S)-crizotinib/MTH1 complex, with the averaged RMSDs of 1.35 and 2.48 Å for protein and ligand, respectively. Moreover, fluctuation of the active site for (R)-crizotinib is more significant than that for (S)-crizotinib.

The RMSFs from the initial structure of the MTH1 backbone atoms over the MD simulations is shown in Fig 2D. The RMSF analysis of each residue highlighted that the loop connecting the residues 23–32, 38–44, 88–101 and140-144 is the most flexible region.

It is worth noting that the side chains of some important residues like Phe27 and Phe139 adopt different conformation when compared with the X-ray structure. S1 Fig shows the superposition of the stable structure extracted from equilibrium trajectories with the X-ray structure. It can be seen that the backbone of stable MTH1 protein do not deviate much from the X-ray structure in the two complexes. As for the force field for the simulation of crizotinib, the works by Sun et al [41, 42] proved the successful use of GAFF force field for the simulation of crizotinib.

### Binding free energy calculations by MM/GBSA and ABF

The predicted binding free energies (Δ*G*_bind_) and the energy components of the (S)-crizotinib/MTH1 and (R)-crizotinib/MTH1 complexes computed by MM/GBSA are summarized in Table 1. The binding affinity of the (S)-crizotinib/MTH1 complex (-24.77 kcal/mol) predicted by MM/GBSA is obviously lower than that of the (R)-crizotinib/MTH1 complex (-14.60 kcal/mol). The order of the calculated binding free energies is consistent with that of the experimental IC_50_ data. According to Table 1, electrostatic energy(ΔE_ele_) and van der Waals energy(ΔE_vdw_) terms in the gas phase provide the major favorable contributions to the (S)-crizotinib and (R)-crizotinib MTH1 binding, whereas polar solvation energies (ΔG_GB_) and -TΔS impair the binding. Furthermore, the nonpolar contribution (-52.84 kcal/mol), which is the sum of the ΔE_ele_ and ΔE_vdw,_ for (S)-crizotinib binding is obviously favorable than that (-50.69 kcal/mol) for the (R)-crizotinib binding. The van der Waals term, is more crucial than the electrostatic part for determining the chirality selectivity of crizotinib. As MM/GBSA calculations do not include entropic terms, we estimated the corresponding entropic contributions (-TΔS) upon binding of ligands and MTH1. The values are 15.07 kcal/mol for (S)-crizotinib/MTH1 complex and 21.62 kcal/mol for (R)-crizotinib/MTH1 complex suggesting that conformational change also plays an important role to protein-ligand interaction.

**Table 1:** The binding free energies and individual energy terms of (S)-crizotinib and (R)-crizotinib to MTH1 predicted by MM/GBSA.

	(S)-crizotinib/MTH1	(R)-crizotinib/MTH1
Contribution	Mean (kcal/mol)	Mean (kcal/mol)
**Δ*E***_**ele**_	-154.17±0.80^c^	-189.30±1.50
**Δ*E***_**vdw**_	-46.43±0.03	-44.16±0.12
**Δ*G***_**SA**_	-6.41±0.12	-6.53±0.10
**Δ*G***_**GB**_	167.17±0.74	203.77±0.01
**Δ*G***_**nonpolar**_ ^***a***^	-52.84±0.25	-50.69±0.20
**Δ*G***_**polar**_ ^***b***^	13.00±0.07	14.47±0.11
**Δ*G***_**total,GB**_	-39.84±0.29	-36.22±0.41
**-TΔS**	15.07±0.21	21.62±0.47
**Δ*G***_**bind,GB**_	-24.77±0.36	-14.60±0.62

^*a*^ *G*_nonpolar_ = Δ*E*_vdw_+Δ*G*_SA_

^*b*^ *G*_polar_ = Δ*E*_ele_ +Δ*G*_GB_.

^C^ standard deviations calculated through 10 times of repeated sampling from 10-20ns trajectory.

Table 2 illustrates that the binding free energies based on the PMF depth (ΔW_PMF_) are the same with those respect to the corresponding system. It can be found that the ranking of the binding free energies predicted by these two protocols all agree well with the experimental IC_50_ data. Here, we estimate the standard deviation of the free energy by MM/GBSA and ABF methods by repeating multiple trajectories (Table 2), and we have reason to believe that this error may be caused by the different force fields used in the two protocols (the MM/GBSA method uses implicit solvent and the ABF method uses explicit solvent, respectively). Since the correct ranking of the binding free energies is usually emphasized in molecular designs, it is reasonable to accept the results above.

**Table 2:** Binding free energies of (S)-crizotinib/MTH1 and (R)-crizotinib/MTH1 complexes by MM/GBSA and ABF.

Name	(S)-crizotinib/MTH1	(R)-crizotinib/MTH1
**ΔG**_**bind**_ **(kcal/mol)**	-24.77±0.36	-14.60±0.62
**ΔG**_**PMF**_ **(kcal/mol)**	-29.60±0.38^a^	-22.38±0.51
**IC**_**50**_ **(nM)**	72	1375

^a^ standard deviation calculated based on 18-23Å reaction coordinates of the 5 replicas we simulated.

We also calculated the strain energy [48] (the changes of the enthalpy of the ligand translating from the unbound conformation to bound conformation, calculated by MM-GBSA method) of (S)-crizotinib and (R)-crizotinib, the strain energy of (S)-crizotinib (1.08 kcal/mol) is lower than that of (R)-crizotinib (5.69 kcal/mol). The fluctuation of (R)-crizotinib heavy atom is larger than that of (S)-crizotinib (Fig 2C).

### Decomposition of effective energies on a per residue basis

**Fig 3:**
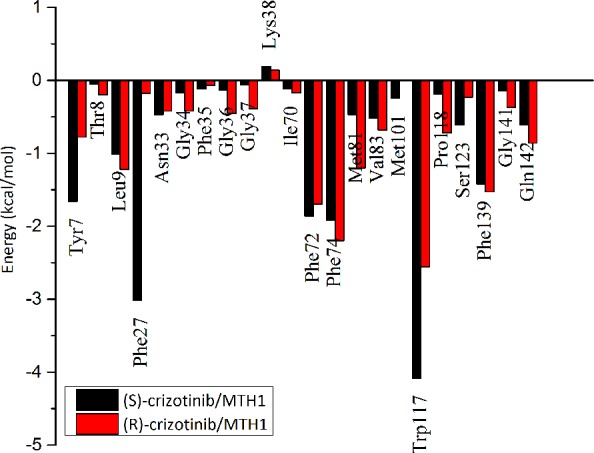
Intermolecular ligand receptor (MTH1) interaction spectrum of the MTH1-crizotinib complex according to the MM/GBSA analysis methods.

**Fig 4:**
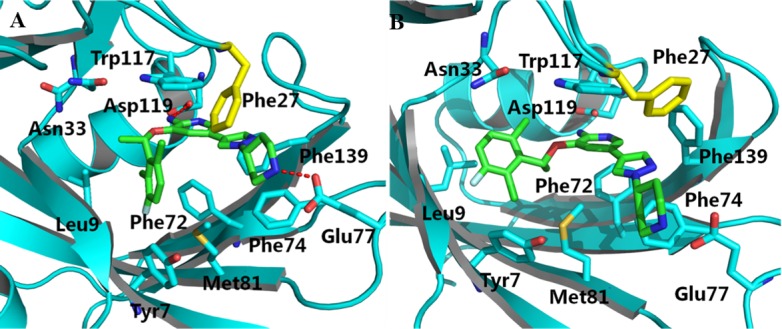
MD-simulated structures of (S)-crizotinib (A) and (R)-crizotinib (B) bound to the active site of the MTH1 protein. The protein is represented as blue cartoon, respectively. Blue stick representations are shown for residues in the active site. The green stick is the ligand. The important residue is illustrated in yellow stick in MTH1 protein. Red dashed lines represent the hydrogen bond.

The binding free energies in the (S)-crizotinib/MTH1 or (R)-crizotinib/MTH1 complexes was decomposed into contribution of residues by using MM/GBSA approach. Comparison of the interactions spectra (Fig 3 and Table 3) shows that the selective binding between (S)-crizotinib and (R)-crizotinib to MTH1 is primarily determined by Tyr7, Phe27, Phe72 and Trp117. The contribution of Phe27 to the binding of (S)-crizotinib (-3.02 kcal/mol) is substantially stronger than that to (R)-crizotinib (-0.18 kcal/mol). Structural analysis provides a more deep insight into the basis of the selectivity (Fig 4). The conformation of the phenyl group of the side chain of Phe27 in the (R)-crizotinib/MTH1 complex is significantly different from that in the (S)-crizotinib/MTH1 complex. The phenyl group of the side chain of Phe27 in the (R)-crizotinib/MTH1 complex is far from the piperidine ring of (R)-crizotinib, implying that Phe27 forms more favorable contacts with (S)-crizotinib than (R)-crizotinib. To be more specific, this structural difference might explain the difference of the van der Waals interactions. For residue Trp117, its contribution to the (R)-crizotinib and (S)-crizotinib bindings are -2.56 kcal/mol and -4.09 kcal/mol, respectively. The favorable free energy contribution of Trp117 for (S)-crizotinib is mainly from the van der Waals interaction (-4.24 kcal/mol).

**Table 3:** The contributions of the important residues for the ligand binding predicted by the MM/GBSA free energy decomposition (kcal/mol).

	(S)-crizotinib/MTH1				(R)-crizotinib/MTH1			
Residue	Δ*E*_vdw_	Δ*E*_ele_	Δ*G*_sol_ ^*a*^	Δ*G*_total_ ^*b*^	Δ*E*_vdw_	Δ*E*_ele_	Δ*G*_sol_	Δ*G*_total_
**Tyr7**	**-1.90**	**-0.40**	**-0.17**	**-1.66**	**-0.88**	**0.28**	**-0.18**	**-0.78**
**Thr8**	-0.10	-0.23	0.27	-0.05	-0.21	-0.48	0.48	-0.20
**Leu9**	-0.93	0.17	-0.26	-1.01	-1.15	0.34	-0.42	-1.22
**Phe27**	**-2.49**	**-0.50**	**-0.03**	**-3.02**	**-0.30**	**0.02**	**0.10**	**-0.18**
**Asn33**	-0.88	-0.31	0.72	-0.47	-0.84	0.57	-0.15	-0.42
**Gly34**	-0.21	-0.66	0.70	-0.17	-0.37	-1.17	1.12	-0.42
**Phe35**	-0.10	0.19	-0.21	-0.12	-0.25	0.30	-0.11	-0.07
**Gly36**	-0.14	0.10	-0.09	-0.13	-0.63	-0.06	0.24	-0.45
**Gly37**	-0.09	0.33	-0.30	-0.06	-0.37	-0.01	0.00	-0.39
**Lys38**	-0.04	12.6	-12.3	0.19	-0.07	10.9	-10.7	0.14
**Ile70**	-0.12	-0.24	0.24	-0.12	-0.17	-0.16	0.16	-0.17
**Phe72**	**-1.92**	**0.01**	**0.05**	**-1.86**	**-1.71**	**-0.29**	**0.30**	**-1.70**
**Phe74**	-1.73	0.12	-0.32	-1.92	-2.11	0.15	-0.24	-2.20
**Met81**	-0.49	-0.42	0.44	-0.47	-1.17	-0.05	0.01	-1.20
**Val83**	-0.43	-0.05	-0.05	-0.52	-0.62	-0.07	0.01	-0.68
**Met101**	-0.26	0.45	-0.43	-0.24	-0.02	0.29	-0.28	-0.01
**Trp117**	-4.24	0.26	-0.11	-4.09	-2.61	0.24	-0.18	-2.56
**Pro118**	-0.18	0.41	-0.42	-0.19	-0.64	0.30	-0.38	-0.72
**Ser123**	-0.73	0.14	-0.02	-0.61	-0.22	0.48	-0.49	-0.23
**Phe139**	-1.52	0.11	0.20	-1.42	-1.48	-0.39	0.34	-1.53
**Gly141**	-0.49	-2.04	2.39	-0.14	-0.47	-2.60	2.70	-0.37
**Gln142**	-1.00	-0.13	0.53	-0.16	-2.23	-2.71	4.08	-0.86

^*a*^Δ*G*_sol_: Desolvation energy of residue to ligand binding

^*b*^Δ*G*_total_: Total contribution of residue to ligand binding.

Difference in the binding free energies also occurs in Tyr7, with -1.66 and -0.78 kcal/mol for (S)-crizotinib and (R)-crizotinib. It can also see that the conformational change of the 2,6-dichloro-3-fluoro-phenyl of the (R)-crizotinib comparing to that of (S)-crizotinib and the conformational change of Tyr7 in the (R)-crizotinib/MTH1 complex is larger than that in the (S)-crizotinib/MTH1 complex (Fig 2D). This is also in accordance with the fact that the favorable contribution is mainly from the van der Waals energy. It also further illustrates that Tyr7, Phe27, Phe72 and Trp117 are key residues in selective binding.

### Comparison of the PMFs along the Reaction Coordinates

**Fig 5:**
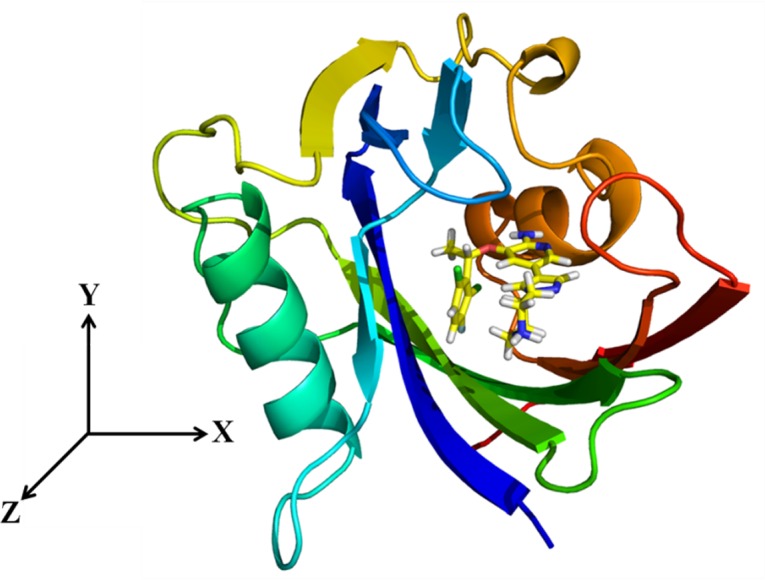
The MTH1 active pocket and the Z-axis is the direction of the ligand escape from the active pocket of MTH1.

**Fig 6:**
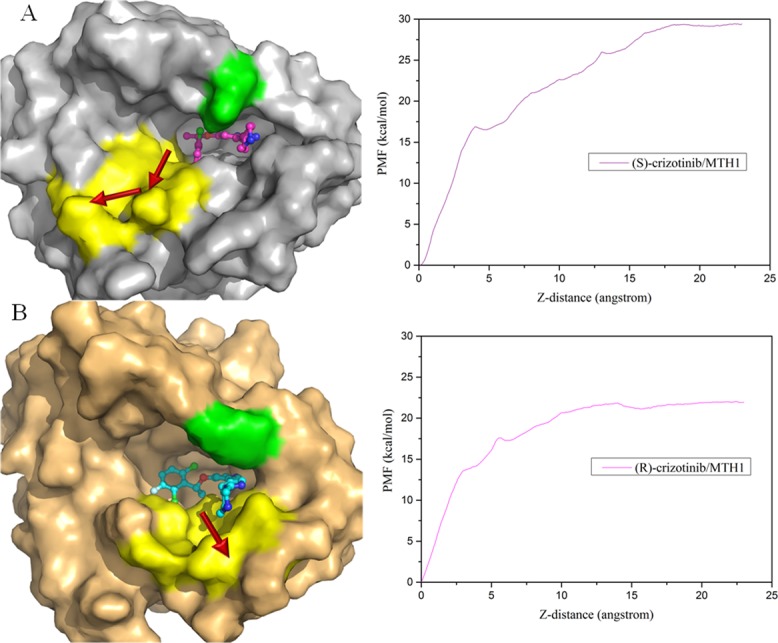
Initial structures of the ABF and the PMF curves of the (S)-crizotinib complex (A) and (R)-crizotinib complex (B). Red arrows represent the direction of the crizotinib escaping from the binding pocket. The yellow region denotes the necessary bin ding channel for (R)-crizotinib escaping out of the active pocket, and the green region represents the important residue Phe27 for the binding of (R)-crizotinib.

As shown in Fig 5, the direction of the largest pocket was chosen as the RC, and it was rotated along Z-axis for the projection of the biasing force. The beginning of the RC for the determination of the PMF changes was defined as the distance between the center of mass of the ligand and the center of mass of the backbone of MTH1. During the simulations, the RC spanned to ~23.0 Å. Fig 6 shows the initial structures of the (S)-crizotinib/MTH1 and (R)-crizotinib/MTH1 complexes, and their corresponding PMF curves for the crizotinib unbinding process from the ABF simulations.

**Fig 7:**
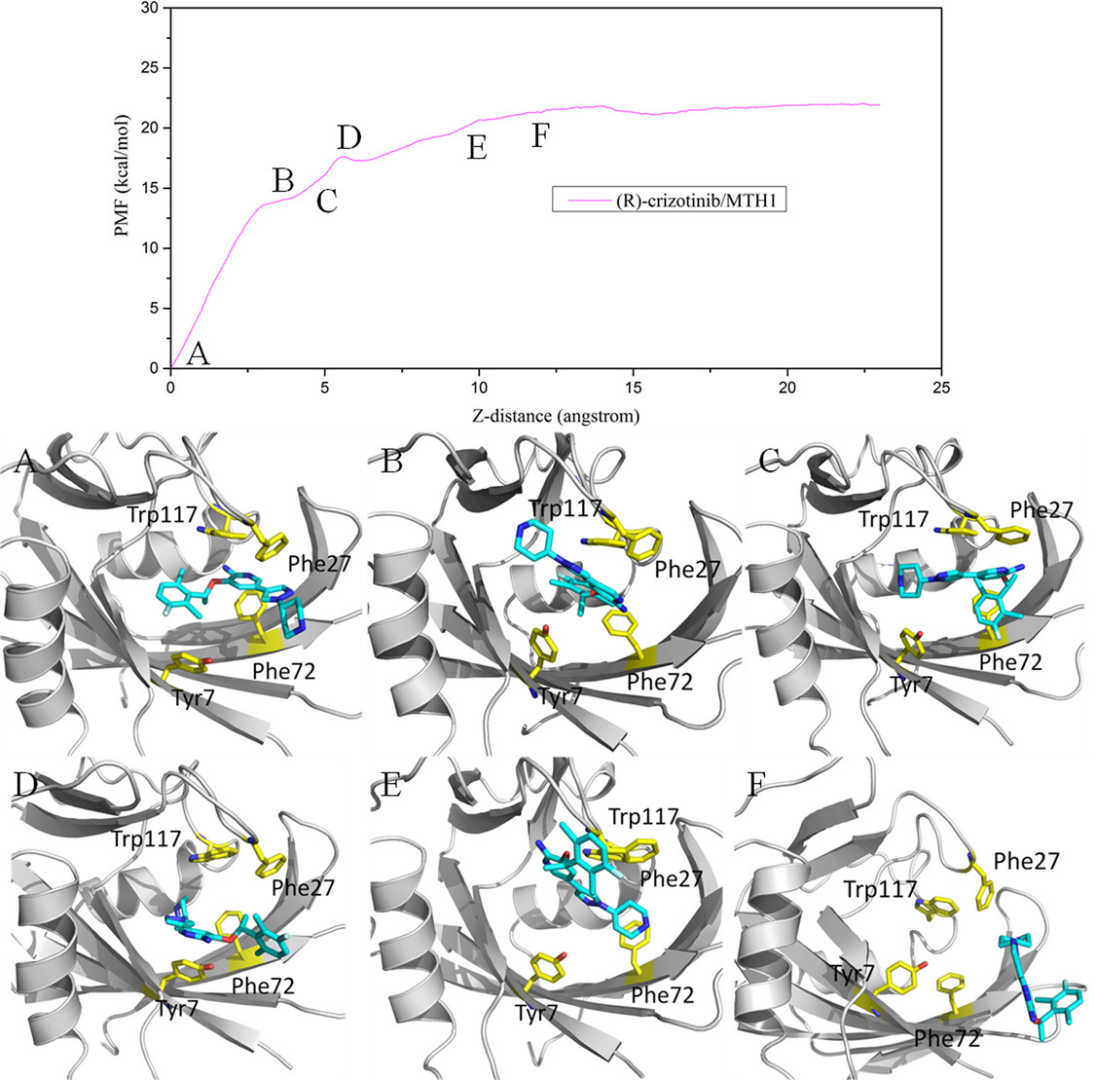
Conformation of (R)-crizotinib along the reaction coordinate (Z-axis) in the MTH1 protein. Top: the PMF along the reaction coordinate. Bottom: Corresponding representatives’ structures of the (R)-crizotinib/MTH1. The proteins are shown in gray cartoon, respectively. Yellow stick representations are shown for important residues. The blue stick is the ligand.

**Fig 8:**
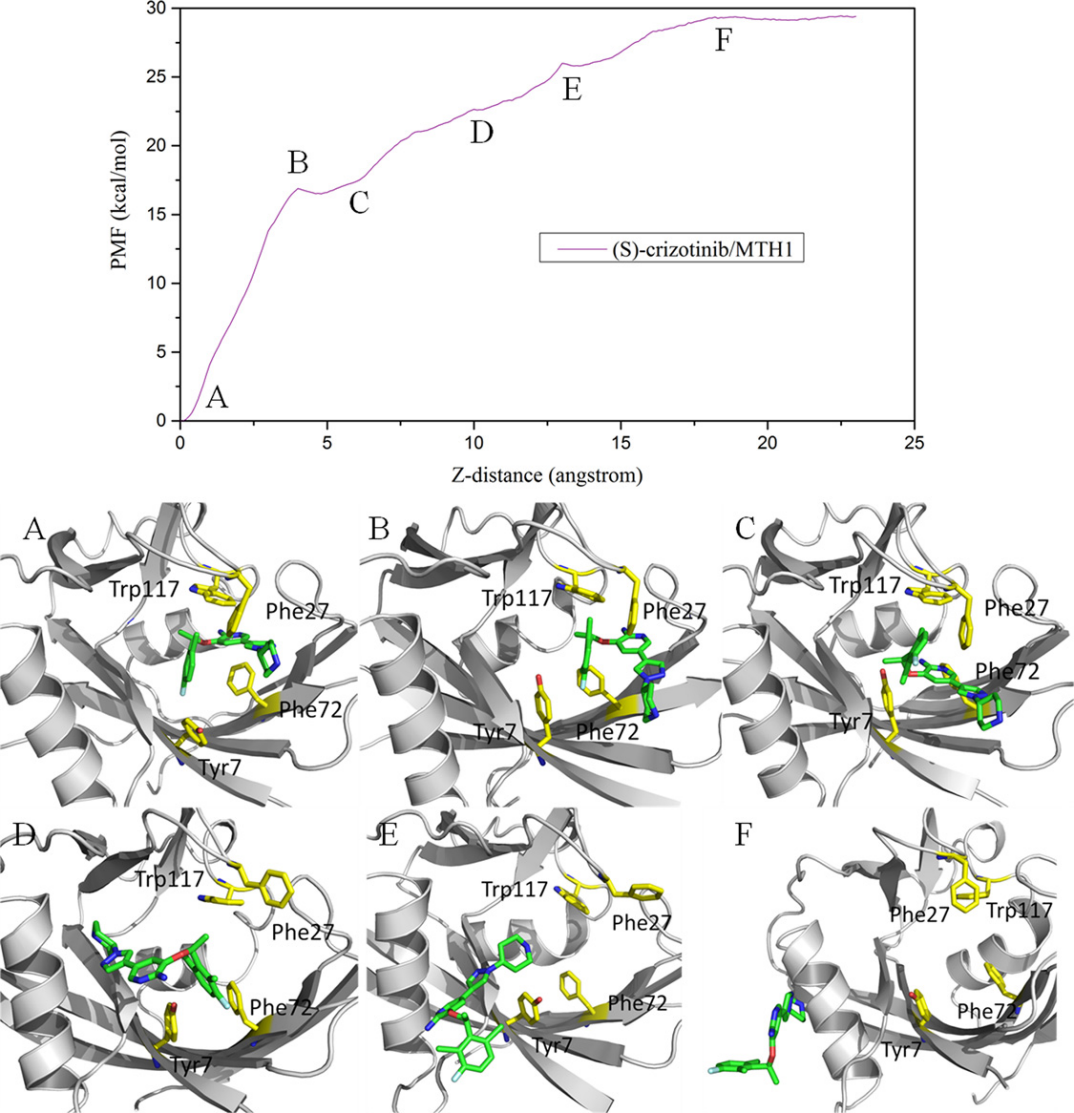
Conformation of (S)-crizotinib along the reaction coordinate (Z-axis) in the MTH1 protein. Top: the PMF along the reaction coordinate. Bottom: Corresponding representatives’ structures of the (S)-crizotinib/MTH1. The proteins are shown in gray cartoon, respectively. Yellow stick representations are shown for important residues. The green stick is the ligand.

It can be seen that the PMF curves for the two systems are different: a platform of the PMFs was observed at ~12 Å of the RC for (R)-crizotinib, while for (S)-crizotinib, the platform is at ~18 Å. The free energy of unbinding for (S)-crizotinib is about 7 kcal/mol higher than that for (R)-crizotinib. The detailed processes of the (R)-crizotinib and (S)-crizotinib unbinding from the MTH1 are illustrated in Figs 7 and 8. As shown in Fig 7, (R)-crizotinib gradually moves from the binding pocket with the increase of the biasing potential added to (R)-crizotinib. At 12 Å of the RC, the biasing potential reaches the maximum value of ~22 kcal/mol and then becomes relatively stable. From Fig 7A–7E, we can see that (R)-crizotinib turns a somersault and then jumps out of the active pocket, which might be affected by the residues along the reaction direction.

As shown in Fig 8, for the (S)-crizotinib/MTH1 complex, before ~12 Å of the RC, the curve is similar to that of the (R)-crizotinib/MTH1 complex. After that the curve of the (S)-crizotinib/MTH1 complex rises continuously to ~18 Å of the RC around ~29 kcal/mol, while the (R)-crizotinib/MTH1complex already reaches a platform around ~22 kcal/mol. The gap between these two platforms is ~7 kcal/mol. It can be seen that Phe27 in the (S)-crizotinib/MTH1 complex narrow the entrance of the binding pocket and a V-like route was adopted by (S)-crizotinib to escape out of the binding pocket as shown in Fig 6A.

## Conclusions

In this work, we investigated the influence of chirality of crizotinib on its MTH1 Inhibitory activity by the use of molecular dynamics simulations and binding free energy calculations. The comparison of the PMFs indicates (S)-crizotinib and (R)-crizotinib have different reaction coordinates when they escape from MTH1. The binding free energy calculations from MM/GBSA and ABF calculation method are in good agreement with the experimental data. Residue decomposition shows that the decrease of the binding energies for Tyr7, Phe27, Phe72 and Trp117 is primarily contributed from the conformation rearrangement of the MTH1 active site of the (R)-crizotinib. The residue Phe27 breaks the balance of (R)-crizotinib inside the binding pocket. Our studies are helpful to elucidate the chirality effect of crizotinib inhibition activity on MTH1 protein. These results are valuable for designing new novel MTH1 inhibitor in the future.

## Supporting Information

S1 Fig Superposition of the stable structure extracted from equilibrium trajectory with the X-ray structure. A. (S)-crizotinib/MTH1 protein complex; B. (R)-crizotinib/MTH1 protein complex.
(TIF)

S2 Fig Convergence of the potentials of mean force (PMFs), and it is convergent. (TIF)

S1 Table Atom types and partial charges for (S)-crizotinib. (DOC)

S2 Table Atom types and partial charges for (R)-crizotinib. (DOC)
