# Supplementary figures and images for: Influence of Chirality of Crizotinib on Its MTH1 Protein Inhibitory Activity: Insight from Molecular Dynamics Simulations and Binding Free Energy Calculations
Source: PLoS One. 2015 Dec 17;10(12):e0145219. doi: 10.1371/journal.pone.0145219 (PMC4683072; doi:10.1371/journal.pone.0145219)

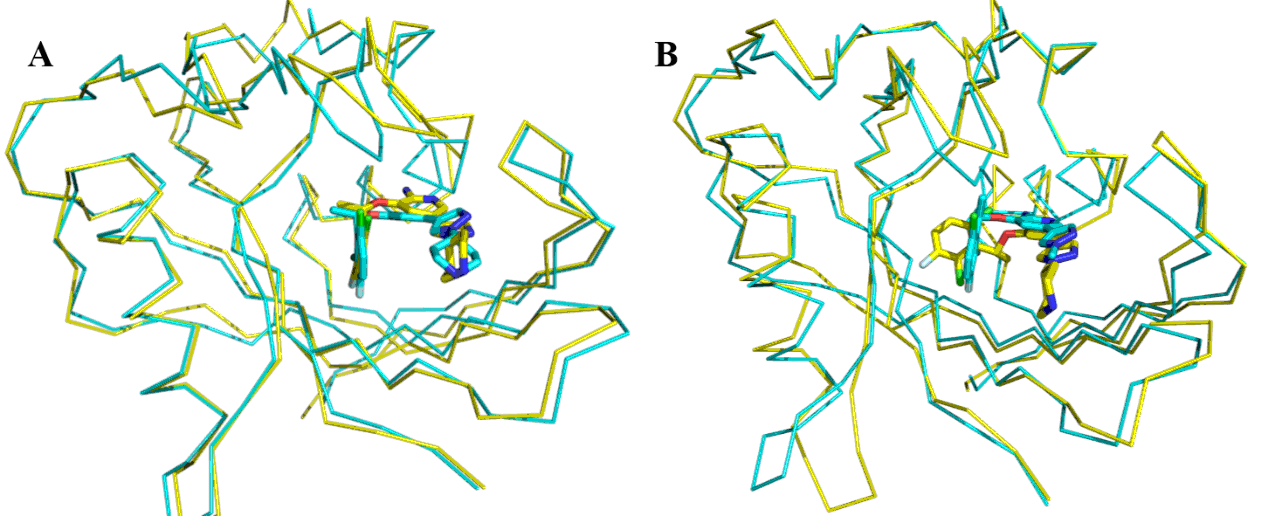

Supplement: S1 Fig — A. (S)-crizotinib/MTH1 protein complex; B. (R)-crizotinib/MTH1 protein complex. (TIF) [file pone.0145219.s001.tif]

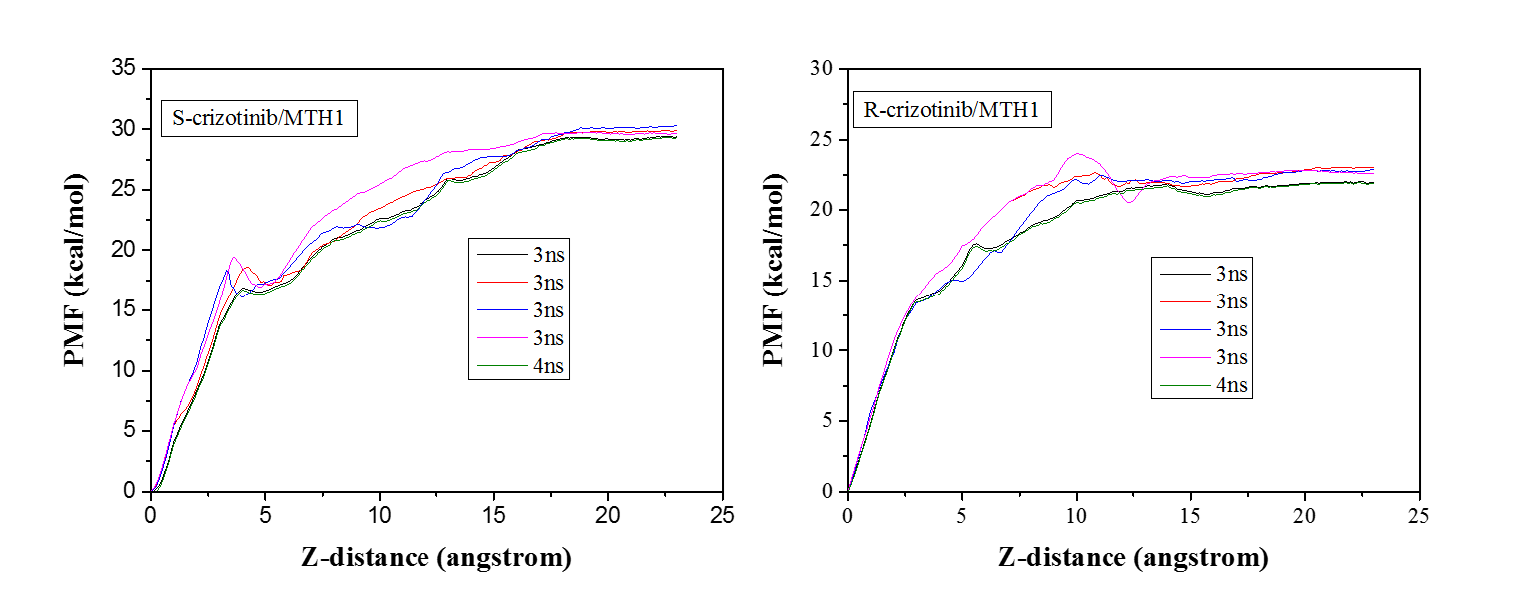

Supplement: S2 Fig — (TIF) [file pone.0145219.s002.tif]
