# Supplementary material for: Influence of Chirality of Crizotinib on Its MTH1 Protein Inhibitory Activity: Insight from Molecular Dynamics Simulations and Binding Free Energy Calculations
Source: PLoS One. 2015 Dec 17;10(12):e0145219. doi: 10.1371/journal.pone.0145219 (PMC4683072; doi:10.1371/journal.pone.0145219)
Supplement: S1 Table — (DOC) [file pone.0145219.s003.doc]

**S1 Table.** Atom types and partial charges for (S)-crizotinib.


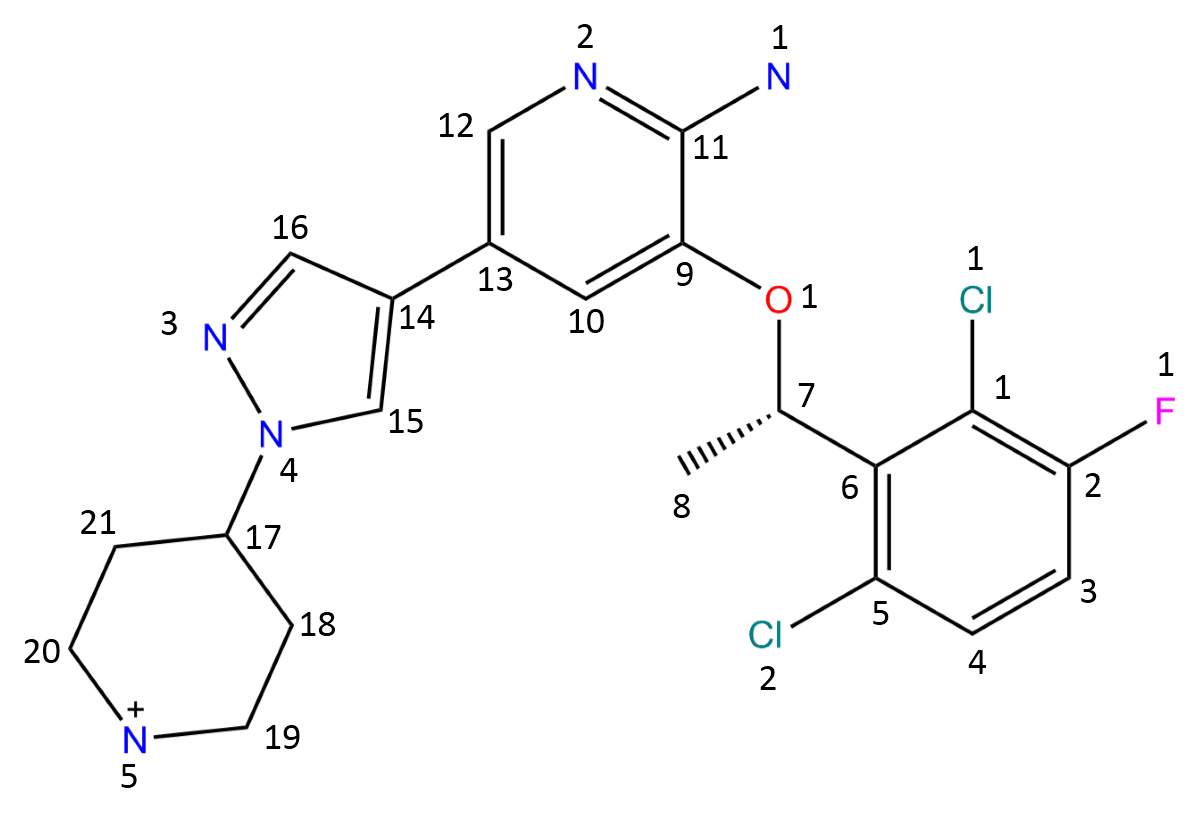


| Atom Name | Atom Type | Partial Charge |
| --- | --- | --- |
| Cl1 | cl | -0.040965 |
| C1 | ca | -0.069647 |
| C2 | ca | 0.294610 |
| F1 | f | -0.178644 |
| C3 | ca | -0.203180 |
| C4 | ca | -0.143829 |
| C5 | ca | 0.024937 |
| Cl2 | c1 | -0.116552 |
| C6 | ca | 0.066568 |
| C7 | c3 | 0.053292 |
| C8 | c3 | -0.165833 |
| O1 | os | -0.246705 |
| C9 | ca | 0.056816 |
| C10 | ca | -0.046490 |
| C11 | ca | 0.551758 |
| N1 | nh | -0.911564 |
| N2 | nb | -0.525741 |
| C12 | ca | 0.101861 |
| C13 | ca | -0.142376 |
| C14 | ce | -0.027386 |
| C15 | c3 | -0.129040 |
| C16 | c2 | 0.062237 |
| N3 | nh | -0.466640 |
| N4 | n3 | 0.127941 |
| C17 | c3 | -0.007966 |
| C18 | c3 | 0.039177 |
| C19 | c3 | -0.085511 |
| N5 | n4 | -0.136991 |
| C20 | c3 | -0.085511 |
| C21 | c3 | 0.039177 |
| H1 | ha | 0.185472 |
| H2 | ha | 0.158424 |
| H3 | h1 | 0.130514 |
| H4 | hc | 0.069636 |
| H5 | hc | 0.069636 |
| H6 | hc | 0.069636 |
| H7 | ha | 0.075985 |
| H8 | hn | 0.402935 |
| H9 | hn | 0.402935 |
| H10 | h4 | 0.117567 |
| H11 | h4 | 0.117567 |
| H12 | h4 | 0.165151 |
| H13 | h1 | 0.073505 |
| H14 | hc | 0.057298 |
| H15 | hc | 0.057298 |
| H16 | hx | 0.109822 |
| H17 | hx | 0.109822 |
| H18 | hx | 0.109822 |
| H19 | hx | 0.109822 |
| H20 | hx | 0.287627 |
| H21 | hc | 0.057298 |
| H22 | hc | 0.057298 |
| H23 | hn | 0.287627 |
