# Supplementary material for: Influence of Chirality of Crizotinib on Its MTH1 Protein Inhibitory Activity: Insight from Molecular Dynamics Simulations and Binding Free Energy Calculations
Source: PLoS One. 2015 Dec 17;10(12):e0145219. doi: 10.1371/journal.pone.0145219 (PMC4683072; doi:10.1371/journal.pone.0145219)
Supplement: S2 Table — (DOC) [file pone.0145219.s004.doc]

**S2 Table.** Atom types and partial charges for (R)-crizotinib.


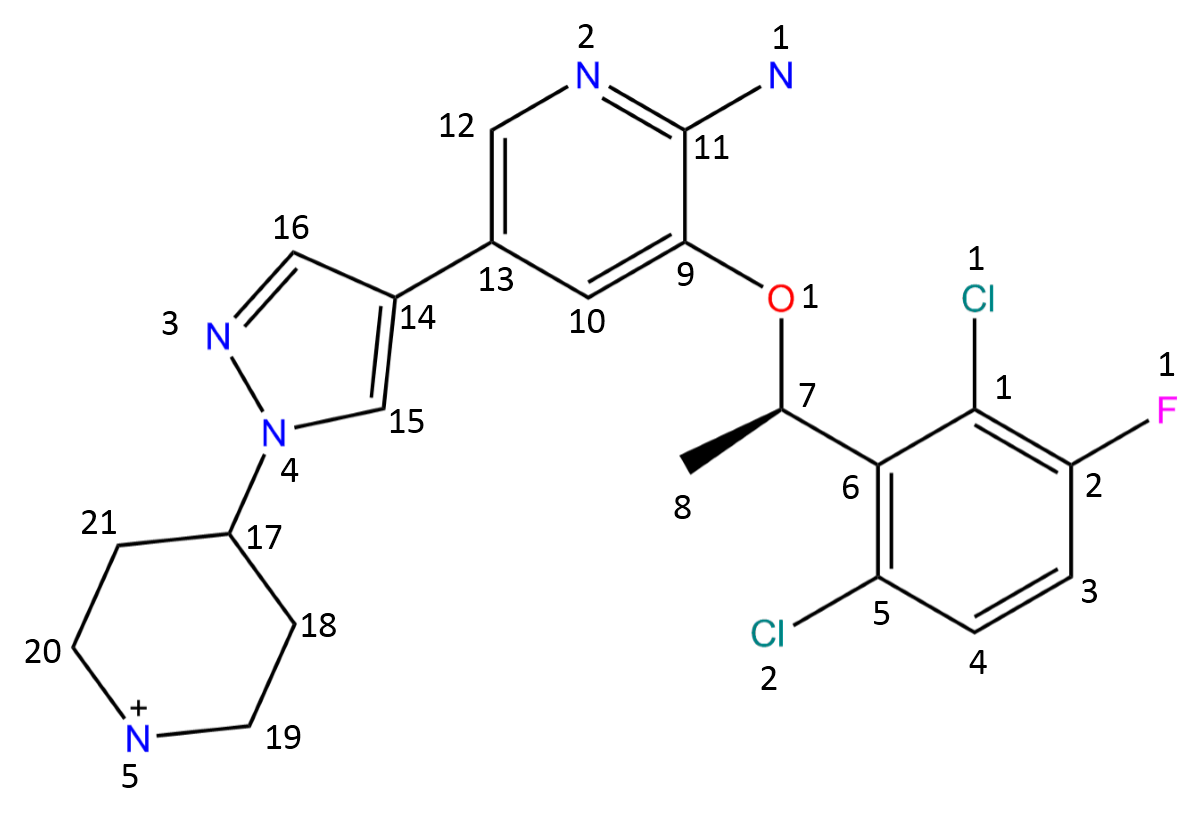


| Atom Name | Atom Type | Partial Charge |
| --- | --- | --- |
| Cl1 | cl | -0.044379 |
| C1 | ca | -0.112507 |
| C2 | ca | 0.292778 |
| F1 | f | -0.171317 |
| C3 | ca | -0.199979 |
| C4 | ca | -0.154875 |
| C5 | ca | 0.026626 |
| Cl2 | c1 | -0.121457 |
| C6 | ca | 0.036564 |
| C7 | c3 | 0.356774 |
| C8 | c3 | -0.174786 |
| O1 | os | -0.454748 |
| C9 | ca | 0.064641 |
| C10 | ca | -0.014694 |
| C11 | ca | 0.582897 |
| N1 | nh | -0.778084 |
| N2 | nb | -0.567057 |
| C12 | ca | 0.151276 |
| C13 | ca | -0.188656 |
| C14 | ce | -0.031735 |
| C15 | c3 | -0.155118 |
| C16 | c2 | 0.095622 |
| N3 | nh | -0.458762 |
| N4 | n3 | 0.092093 |
| C17 | c3 | 0.013823 |
| C18 | c3 | 0.059389 |
| C19 | c3 | -0.087638 |
| N5 | n4 | -0.133156 |
| C20 | c3 | -0.087638 |
| C21 | c3 | 0.059389 |
| H1 | ha | 0.193256 |
| H2 | ha | 0.172887 |
| H3 | h1 | 0.118542 |
| H4 | hc | 0.050406 |
| H5 | hc | 0.050406 |
| H6 | hc | 0.050406 |
| H7 | ha | 0.040260 |
| H8 | hn | 0.350328 |
| H9 | hn | 0.350328 |
| H10 | h4 | 0.114464 |
| H11 | h4 | 0.197101 |
| H12 | h4 | 0.149115 |
| H13 | h1 | 0.060644 |
| H14 | hc | 0.050437 |
| H15 | hc | 0.050437 |
| H16 | hx | 0.108900 |
| H17 | hx | 0.108900 |
| H18 | hx | 0.108900 |
| H19 | hx | 0.108900 |
| H20 | hx | 0.284613 |
| H21 | hc | 0.050437 |
| H22 | hc | 0.050437 |
| H23 | hn | 0.108900 |
